# Safer Prescribing and Care for the Elderly (SPACE): Protocol of a Cluster Randomized Controlled Trial in Primary Care

**DOI:** 10.2196/resprot.9839

**Published:** 2018-04-26

**Authors:** Katharine Ann Wallis, Carolyn Raina Elley, Arier Lee, Simon Moyes, Ngaire Kerse

**Affiliations:** ^1^ Department of General Practice and Primary Health Care University of Auckland Auckland New Zealand; ^2^ School of Population Health University of Auckland Auckland New Zealand

**Keywords:** general practice, safety, prescriptions, multimorbidity, polypharmacy, adverse drug events

## Abstract

**Background:**

High-risk prescribing, adverse drug events, and avoidable adverse drug event hospitalizations are common. The single greatest risk factor for high-risk prescribing and adverse drug events is the number of medications a person is taking. More people are living longer and taking more medications for multiple long-term conditions. Most on-going prescribing occurs in primary care. The most effective, cost-effective, and practical approach to safer prescribing in primary care is not yet known.

**Objective:**

To test the effect of the Safer Prescribing And Care for the Elderly (SPACE) intervention on high-risk prescribing of nonsteroidal anti-inflammatory and antiplatelet medicines, and related adverse drug event hospitalizations.

**Methods:**

This is a protocol of a cluster randomized controlled trial. The clusters will be primary care practices. Data collection and analysis will be at the level of patient.

**Results:**

Recruitment started in 2018. Six-month data collection will be in 2018.

**Conclusions:**

This study addresses an important translational gap, testing an intervention designed to prompt medicines review and support safer prescribing in routine primary care practice.

**Trial Registration:**

Australian New Zealand Clinical Trials Registry: ACTRN12618000034235 http://www.ANZCTR.org.au/ACTRN12618000034235.aspx (Archived with Webcite at http://www.webcitation.org/6yj9RImDf)

## Introduction

### Avoidable Adverse Drug Events

Adverse drug events (ADEs) and avoidable ADE hospital admissions are common, costing health systems billions of dollars every year [1-7]. Internationally, approximately 7% of hospital admissions result from drug-related problems, of which 59% are considered avoidable through safer prescribing [3,4,8]. Most drug-related admissions are caused by commonly prescribed drugs, notably nonsteroidal anti-inflammatory drugs (NSAIDs), antiplatelet medications, and anticoagulants, which together account for one-third of ADE admissions [3,4,9].

### High-risk Prescribing

High-risk prescribing is prescribing that places patients at increased risk of ADEs. The single greatest predictor of ADEs and high-risk prescribing is the number of medications a person is taking [10]. With demographic ageing, there are increasing numbers of older people prescribed multiple medications for multiple co-existing medical conditions [11]. In New Zealand, approximately 10% of people aged 65 years and older are taking ten or more regular medications, and high-risk prescribing is common, often involving NSAIDs [12-14]. The individual circumstances of a patient may justify high-risk prescribing, but to minimize harm it is necessary that medications are regularly reviewed and stopped or started as appropriate [15].

Most on-going medications are prescribed in primary care. Despite strong evidence to guide safe prescribing, a gap remains between existing evidence and current prescribing practice. Translating research evidence into practice is difficult. There are many barriers to regular medication review in everyday practice [16]. The large variation in prescribing between practices and regions in New Zealand suggests room for improvement [13,17,18].

### Safer Prescribing

In New Zealand primary care, most quality improvement processes are delivered through Primary Health Organisations (PHOs), professional groupings of practices for administrative and quality improvement purposes [19]. The most effective, cost-effective, and practical approach to safer prescribing in everyday practice is not yet known [20,21]. There is evidence to suggest education programmes can improve prescribing but education alone is not enough to induce lasting change [13,22]. Complex interventions as part of ongoing quality improvement programs show the most promise, in particular interventions combining audit and feedback, education, incentive for participation, and patient engagement [20,23-26]. The Australian Veterans’ Medicines Advice and Therapeutics Education Service (MATES) quality improvement program in primary care has shown promising results in the Australian Veterans population, especially when delivering a focused message targeting single medications and less so when delivering a combination of messages targeting general topics such as interactions and potentially inappropriate medications in older people with polypharmacy [24]. The MATES programme is based on sound theoretical underpinnings and delivers 4 interventions per year. The MATES intervention uses practice prescribing audits, patient-specific feedback, education to doctors, and a practice mail-out to selected at-risk patients to encourage their engagement.

### The Safer Prescribing and Care for the Elderly (SPACE) Intervention

Adapted from the Australian Veterans’ MATES programme, we developed the Safer Prescribing and Care for the Elderly (SPACE) intervention to prompt medication reviews and support safer prescribing in the New Zealand primary care context. We recently piloted the SPACE intervention in two New Zealand primary care practices in preparation for this proposed randomized trial, focusing on the topic of NSAIDs and antiplatelet prescribing. This topic was chosen because these drugs are commonly prescribed and are associated with serious ADEs including bleeding and renal impairment. The SPACE intervention was found to be feasible to implement using existing primary care structures and both acceptable and useful to patients, doctors, and the PHO clinical advisory pharmacists [27]. In the pilot study, we developed practice audit queries to identify patients with high-risk prescribing of NSAIDs and/or antiplatelet medications; integrated the SPACE intervention into practice management software; developed processes to collect, encrypt, and link study data; and derived information for calculating sample sizes for the randomized trial.

### Objectives

We will assess whether the SPACE intervention can reduce the rate of high-risk prescribing of NSAIDs and/or antiplatelet medications and related ADE hospitalizations over 12 months using existing PHO infrastructure and systems in New Zealand primary care.

### Trial Design

We will conduct a cluster randomized control trial. The clusters will be primary care practices. Data collection and analysis will be at the level of patient.

## Methods

### Setting

The study will be conducted in primary care practices in Auckland and Northland, New Zealand.

### Eligibility Criteria

Primary care practices will be eligible to participate if:

1. The practice is based in the Auckland or Northland region of New Zealand and has not recently participated in a similar NSAID audit exercise or taken part in the pilot for the SPACE trial
2. The practice uses electronic practice management software compatible with our data collection systems
3. The practice has fewer than 15,000 enrolled patients
4. All physicians in the practice consent to participate

The study will target all physicians working in participating practices since patients can receive a prescription from any physician in a practice.

Patient inclusion criteria:

1. Patients of any age (“vulnerable patients”) will be included in the study if, at baseline, they fulfil one or more of the following inclusion criteria as listed in Table 1 that puts them at increased risk of an ADE related to NSAIDs and/or antiplatelet medications.
2. Participants are “vulnerable patients” at baseline; that is, those patients at increased risk of gastrointestinal, renal or cardiac adverse events related to NSAIDs and/or antiplatelet medications (Table 1).

**Table 1:** Categories of vulnerable patients and high-risk prescribing of NSAIDs and antiplatelet medications [23]. ADE: adverse drug event.

Type of adverse drug event	Risk factor making patients vulnerable (at increased risk of ADE)	High-risk prescribing
Gastrointestinal	Prior peptic ulcer	In patient with prior peptic ulcer, NSAID or aspirin without gastro-protection
	75 years and older	In patient 75 years and older, NSAID without gastro-protection
	65 years and older prescribed aspirin	In patient 65 years and older taking aspirin, NSAID without gastro-protection
		In patient 65 years and older taking aspirin, clopidogrel without gastro-protection
	Prescribed oral anticoagulant	In patient taking an oral anticoagulant, NSAID without gastro-protection
		In patient taking an oral anticoagulant, aspirin or clopidogrel without gastro-protection
Renal	Prescribed both renin-angiotensin system blocker and diuretic	In patient taking both renin-angiotensin system blocker and diuretic, NSAID
	Chronic kidney disease (Estimated Glomerular Filtration Rate[eGFR] <60)	In patient with chronic kidney disease (eGFR <60), NSAID
Cardiac	Heart failure	In patient with history of heart failure, NSAID

All data collected on patients is anonymized prior to leaving the practice using a unique identifier (National Health Index number) to enable linking of clinical data over time and linking to hospitalization data.

### Intervention

The SPACE intervention is designed to prompt medication reviews and support safer prescribing decisions in primary care practice. The intervention comprises a practice audit to identify for each doctor a list of their patients with high-risk prescribing for the chosen topic; an outreach visit from a clinical advisory pharmacist to physicians to provide education about the prescribing topic and to go through with each physician their list of patients identified as having high-risk prescribing; a tick-box for physicians to indicate the action they will take in response to the feedback for each patient (“review medications + patient mail-out,” “review medications + no mail-out,” or “no action”); and a mail-out from the practice to patients selected by physicians with information about their medications and a letter encouraging them to discuss their medications when they are at the practice next seeing their physician [27]. All prescribing decisions are made as usual by the doctor in discussion with the patient. The individual circumstances of the patient may justify high-risk prescribing and, after review, the prescribing may or may not be changed. The prescribing topic for the trial is the prescribing of NSAIDs and antiplatelet medications.

Control practices will deliver care as usual. If the SPACE intervention is shown to be effective, we aim to deliver the intervention to control practices after 12 months.

**Figure 1:**
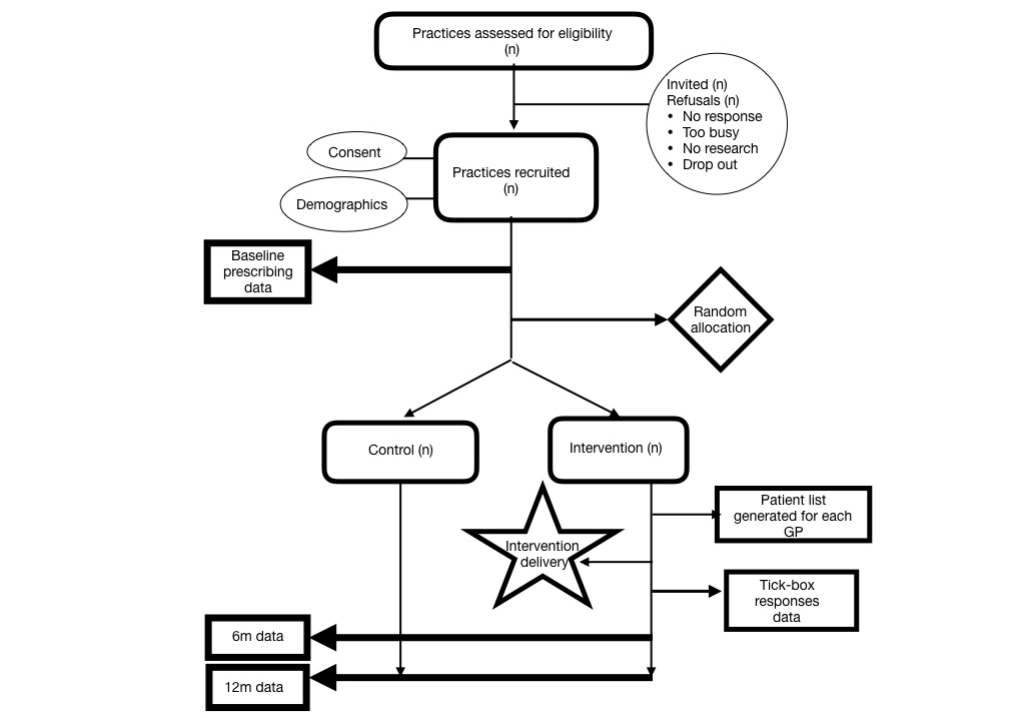
Flow of practices through the randomized control trial.

All practices and doctors will participate as usual in PHO quality improvement initiatives and medical education activities. See Figure 1 for the flow of practices through the study.

### Outcome Measures

Assessment time-points will be baseline, 6 months, and 12 months. The outcomes of interest are the difference between intervention and control groups at 6 months controlling for baseline, and the difference between intervention and control groups at 12 months.

The primary outcome measure is:

The difference in proportion of the participants (those vulnerable at baseline) receiving high-risk prescribing of NSAID and/or antiplatelet medications between the control and intervention groups at 6 months. That is, the proportion of “vulnerable-at-baseline patients” (with gastro-intestinal, renal, or cardiac risk factors) receiving high-risk prescribing of NSAID and/or antiplatelet medications at 6 months according to the definitions listed in Table 1. Whether difference in proportion between the two groups is sustained at 12 months will also be examined. Participants will be considered to have high-risk prescribing at each time-point if they fulfil any of the high-risk prescribing criteria set out in Table 1 in the 14 weeks leading up to each time-point.

Secondary outcome measures include:

1. The difference in proportion of study participants at increased risk of gastrointestinal ADEs according to the definitions listed in Table 1 receiving gastrointestinal high-risk prescribing of NSAID and/or antiplatelet medications between the control and intervention groups at 6 months.
2. The difference in proportion of study participants at increased risk of renal ADEs according to the definitions listed in Table 1 receiving renal high-risk prescribing of NSAID medications between the control and intervention groups at 6 months.
3. The difference in proportion of study participants at increased risk of cardiac ADEs according to the definitions listed in Table 1 receiving cardiac high-risk prescribing of NSAID medications between the control and intervention groups at 6 months.
4. The difference in proportion of study participants (vulnerable patients), and those with high risk prescribing, admitted for related adverse drug events (gastrointestinal ulcer or bleeding, acute kidney injury, and heart failure) between the control and intervention groups during the 12 months after baseline for the intervention. Hospitalization data will be linked to primary care patient data by encrypted National Health Index.
5. The difference in proportion of vulnerable patients in the practice overall receiving high-risk prescribing of NSAIDs and/or antiplatelet medications between the control and intervention groups at 6 months. This will include newcomers to the practice and practice patients who were not vulnerable at baseline but were at 6 months and/or 12 months.

Whether difference in proportion between the 2 groups is sustained at 12 months will also be examined for secondary outcomes 1, 2, 3, and 5 months.

These outcomes have been used in similar trials previously [23]. Data will also be collected to enable a subsequent cost-effectiveness evaluation of the intervention from a societal and health funder perspective. The cost-effectiveness of the intervention will be measured as the cost per reduction in high-risk prescribing and cost per reduction in hospitalizations from the health funder (District Health Board) perspective. The data collected will include the cost of delivering the intervention (including pharmacist and doctor time for the feedback outreach session, travel time and costs for the outreach visit, audit time and cost); cost of medications; cost of hospitalizations.

### Sample Size

The sample size calculation for this study is based on previous trials demonstrating a clinically relevant 25-45% relative risk reduction in the proportion of high-risk prescribing, [23,25] one trial of which also demonstrated how such reduction (3.7% to 2.2%) in high-risk prescribing can translate to significant reductions in hospitalizations due to bleeding complications [23]. Based on the local pilot data, we estimated an average of 200 “vulnerable patients” per practice and an 8% high-risk prescribing rate [28]. We estimated an intracluster correlation coefficient of 4.68 x 10^-7^ for the primary outcome based on a cluster randomized trial examining similar outcome of NSAIDs prescribed to patients with a history of peptic ulcer and not prescribed gastro-protection [25]. Assuming approximately 12% of patients would be lost to follow-up over the 12 month study period, data from 8000 patients from 40 practices (20 practices in each group) with an average of 200 vulnerable patients per practice would be required to detect a statistically significant difference of 6% in the intervention group and 8% in the control group of high-risk prescribing at 12 months (*P*=.90, alpha=.05).

### Recruitment

Practices will be purposively sampled and recruited, aiming to include both medium sized (3000-7999 enrolled patients) and smaller practices (0-2999 enrolled patients) in the Auckland and Northland regions. Practices and physicians will be invited by a colleague using a comprehensive list of practices. Consent for participation will be at the practice and physician level. Written informed consent will be obtained from all participating physicians. Participants, ‘vulnerable patients’, will be identified using a standard query applied to the practice enrolled population. Consent will not be sought from individual patients because outcomes data are collected in routine patient care and will be anonymised prior to extraction for analysis and linking.

### Assignment of Interventions: Randomization

Practices will be randomized 1:1 to intervention or control. Randomization will be stratified by practice location (Auckland vs Northland) and practice size (medium [3000-7999 enrolled patients] and smaller practices [0-2999 enrolled patients]). Block randomization will be carried out using randomly varying block sizes of 2, 4, and 6. Random sequence generation and allocation of randomization will be undertaken by a statistician not involved in recruitment or baseline data extraction.

### Analyses

Analyses will be performed according to the intention-to-treat principle, with the use of mixed-effect models to account for clustering in the data. The primary and secondary outcomes will be analyzed using generalized linear mixed effect model, GLIMMIX, with the individual as the unit of analysis and the practice included as the random effect to control for the effects of clustering. GLIMMIX with Group x Time interaction will be used to assess the overall difference between intervention and control. The model will adjust for the stratification factors including practice location (Northland or Auckland) and practice size. Baseline covariates including (age, sex, and baseline number of long-term medications) will be adjusted if appropriate.

### Ethics

Study approved by the University of Auckland Human Participants Ethics Committee: Ref 020092, expires 9 Oct 2020.

## Results

Recruitment will start in 2018. The SPACE trial will run for 2 years from recruitment to analyses and dissemination.

## Discussion

Most ongoing prescribing occurs in primary care. The prevalence of high-risk prescribing and avoidable ADE admissions, and the unnecessary cost imposed on an already stretched health system, justify greater efforts to improve the safety of prescribing in primary care. The ageing population, with more people living longer and taking more medications for more chronic conditions, means the problems of high-risk polypharmacy and avoidable ADE hospital admissions will continue to increase unless we can improve the safety of prescribing in primary care. The most effective and cost-effective intervention to support safer prescribing in everyday primary care practice is not yet known.

### Intervention Design

The SPACE intervention is based on sound theoretical underpinnings, is acceptable and useful in the New Zealand primary care context, and identifies and reaches patients with high-risk prescribing who are at increased risk of ADEs. The intervention builds on existing primary care infrastructure and uses existing primary care staff to deliver a safety improvement intervention. The intervention combines audit and feedback with mail-out to motivate patient engagement. It is amenable to repeat use, and could be used in an on-going quality improvement program to target different high-risk prescribing topics.

### Practical Applications From Study Results

If shown to be effective and cost-effective, the SPACE intervention could be rolled out nationally and used regularly by PHOs to support safer prescribing in practices and minimize avoidable ADE hospital admissions in the short-to-medium term. Since the SPACE intervention is designed to support behavior change, it could be applied to other evidence-based topics, including other prescribing topics and test ordering and monitoring.

### Study Design

Interventions that have been shown to improve practice have been published [23,25]. However, most previous trials of primary care interventions in this area have involved time-series or noncontrolled trials [20,22,24]. The SPACE trial uses a cluster randomized controlled trial design to provide robust evidence to assess whether such an intervention can change prescribing and improve clinical outcomes.

### Outcome Measures

Since the SPACE intervention is designed to prompt medication reviews and support safer prescribing, the primary outcome measure is designed to reflect a change in prescribing behavior (a reduction in the rate of high-risk prescribing in patients vulnerable at baseline). Since the ultimate aim is to change prescribing behavior overall and improve patient outcomes, we will also measure the rate of high-risk prescribing overall and related ADE hospital admissions.

### Anticipated Challenges

There is a risk of contamination between intervention and control practices and between intervention practices and those waiting for the intervention; doctors might change their prescribing behavior if they are alerted to the study prescribing topic. However, rolling delivery of the intervention is the only practical and feasible way to progress this study, given the educational outreach visit component of the intervention and the limitations of our study team. We are also limited to using small to medium sized practices (fewer than 8000 enrolled patients), since all doctors in participating practices must receive one-on-one feedback from the clinical advisory pharmacist at an outreach visit.

### Conclusions

This study addresses an important translational gap, testing an economically sustainable intervention designed to support safer prescribing in routine practice. The new knowledge generated will help to address the most important threat to patient safety in primary care: high-risk prescribing and adverse drug events.

## Appendix

Multimedia Appendix 1 Reviewers' feedback on funding application for study.

## Abbreviations

ADE: adverse drug event
eGFR: estimated glomerular filtration rate
GLIMMIX: generalized linear mixed effect model
MATES: Australian Veterans’ Medicines Advice and Therapeutics Education Service
NSAID: nonsteroidal anti-inflammatory drug
PHO: Primary Health Organisation
SPACE: Safer Prescribing and Care for the Elderly

## References

[1] Taché Stephanie V, Sönnichsen Andreas, Ashcroft D (2011). Prevalence of adverse drug events in ambulatory care: a systematic review. Ann Pharmacother.

[2] Budnitz DS, Lovegrove MC, Shehab N, Richards CL (2011). Emergency hospitalizations for adverse drug events in older Americans. N Engl J Med.

[3] Pirmohamed M, James S, Meakin S, Green C, Scott AK, Walley TJ, Farrar K, Park BK, Breckenridge AM (2004). Adverse drug reactions as cause of admission to hospital: prospective analysis of 18 820 patients. BMJ.

[4] Howard RL, Avery AJ, Slavenburg S, Royal S, Pipe G, Lucassen P, Pirmohamed M (2007). Which drugs cause preventable admissions to hospital? A systematic review. Br J Clin Pharmacol.

[5] Meier F, Maas R, Sonst A, Patapovas A, Müller Fabian, Plank-Kiegele B, Pfistermeister B, Schöffski Oliver, Bürkle Thomas, Dormann H (2015). Adverse drug events in patients admitted to an emergency department: an analysis of direct costs. Pharmacoepidemiol Drug Saf.

[6] Guthrie B, McCowan C, Davey P, Simpson C, Dreischulte T, Barnett K (2011). High risk prescribing in primary care patients particularly vulnerable to adverse drug events: cross sectional population database analysis in Scottish general practice. BMJ.

[7] Gyllensten H, Rehnberg C, Jönsson Anna K, Petzold M, Carlsten A, Andersson Sundell Karolina (2013). Cost of illness of patient-reported adverse drug events: a population-based cross-sectional survey. BMJ Open.

[8] Thomsen LA, Winterstein AG, Søndergaard B, Haugbølle LS, Melander A (2007). Systematic review of the incidence and characteristics of preventable adverse drug events in ambulatory care. Ann Pharmacother.

[9] (2016). Ministry of Health.

[10] Fried T, O'Leary J, Towle V, Goldstein M, Trentalange M, Martin D (2014). Health outcomes associated with polypharmacy in community-dwelling older adults: a systematic review. J Am Geriatr Soc.

[11] Charlesworth C, Smit E, Lee D, Alramadhan F, Odden M (2015). Polypharmacy Among Adults Aged 65 Years and Older in the United States: 1988-2010. J Gerontol A Biol Sci Med Sci.

[12] Nishtala P, Salahudeen M (2015). Temporal Trends in Polypharmacy and Hyperpolypharmacy in Older New Zealanders over a 9-Year Period: 2005–2013. Gerontology.

[13] Love T, Ehrenberg N (2014). Variation and improving services: case studies and key questions.

[14] Narayan S, Nishtala P (2015). Prevalence of potentially inappropriate medicine use in older New Zealanders: a population-level study using the updated 2012 Beers criteria. J Eval Clin Pract.

[15] Scott I, Hilmer S, Reeve E, Potter K, Le Couteur David, Rigby D, Gnjidic Danijela, Del Mar Christopher B, Roughead Elizabeth E, Page Amy, Jansen Jesse, Martin Jennifer H (2015). Reducing inappropriate polypharmacy: the process of deprescribing. JAMA Intern Med.

[16] Wallis K, Andrews A, Henderson M (2017). Swimming Against the Tide: Primary Care Physicians' Views on Deprescribing in Everyday Practice. Ann Fam Med.

[17] (2015). Health Quality & Safety Commission New Zealand.

[18] Tomlin A, Gillies T, Tilyard M, Dovey S (2016). Variation in the pharmaceutical costs of New Zealand general practices: a national database linkage study. J Public Health (Oxf).

[19] Ministry of Health.

[20] Patterson S, Cadogan C, Kerse N, Cardwell C, Bradley M, Ryan C, Hughes C (2014). Interventions to improve the appropriate use of polypharmacy for older people. Cochrane Database Syst Rev.

[21] Duerden M, Avery T, Payne R (2013). Polypharmacy and medicines optimisation: making it safe and sound.

[22] Richards D, Toop L, Graham P (2003). Do clinical practice education groups result in sustained change in GP prescribing?. Family Practice.

[23] Dreischulte T, Donnan P, Grant A, Hapca A, McCowan C, Guthrie B (2016). Safer Prescribing--A Trial of Education, Informatics, and Financial Incentives. N Engl J Med.

[24] Roughead EE, Kalisch ELM, Ramsay EN, Pratt NL, Barratt JD, LeBlanc VT, Ryan P, Peck R, Killer G, Gilbert AL (2013). Bridging evidence-practice gaps: improving use of medicines in elderly Australian veterans. BMC Health Serv Res.

[25] Avery A, Rodgers S, Cantrill J, Armstrong S, Cresswell K, Eden M, Elliott R, Howard R, Kendrick D, Morris C, Prescott R, Swanwick G, Franklin M, Putman K, Boyd M, Sheikh A (2012). A pharmacist-led information technology intervention for medication errors (PINCER): a multicentre, cluster randomised, controlled trial and cost-effectiveness analysis. The Lancet.

[26] Mugunthan K, McGuire T, Glasziou P (2011). Minimal interventions to decrease long-term use of benzodiazepines in primary care: a systematic review and meta-analysis. Br J Gen Pract.

[27] Wallis K, Tuckey R (2017). Safer Prescribing and Care for the Elderly (SPACE): feasibility of audit and feedback plus practice mail-out to patients with high-risk prescribing. J Prim Health Care.

[28] Wallis K, Elley C, Moyes S, Kerse N (2018). Safer Prescribing And Care for the Elderly (SPACE): Pilot Study in General Practice. British Journal of General Practice Open.

