# Supplementary material for: Safer Prescribing and Care for the Elderly (SPACE): Protocol of a Cluster Randomized Controlled Trial in Primary Care
Source: JMIR Res Protoc. 2018 Apr 26;7(4):e109. doi: 10.2196/resprot.9839 (PMC5945992; doi:10.2196/resprot.9839)
Supplement: Multimedia Appendix 1 [file resprot_v7i4e109_app1.pdf]

PROJECT GRANTS<br>Medical Committee Report Form

Auckland Medical Research Foundation
est. 1955

APPLICANTS	Wallis et al
SHORT TITLE	SPACEcluster RCT in general practice

Overall Summary / Key Points: Please use the following topics as a guide.

- ☐ Relevance: Is the concept important, and are the aims appropriate and clearly defined?
- ☐ Quality: Is the application well written? Does the experimental approach have scientific merit?
- ☐ Experience (Relative to opportunity): Is the application to a standard you would expect for the PI's career stage? Do the applicants have good track records, and are they capable of achieving the objectives?
- ☐ Other: Are there minor or major issues with the application and/or applicants?

The proposal is to run an RCT to reduce adverse events from high-risk prescribing in GP practices.
Medication is the major cause of harm in primary care and in NZ in 2005 there were 15,254 admission for adverse drug events. Some if not many (60%) of these are avoidable. Largest risk factor for ADE is number of medications taken and >10 is high risk. In NZ 10% of older individuals are taking more than 10 meds. The MATES programme in Australia is uses prescribing audits, patient-based feedback and education to improve doctor prescribing, as well as activating at-risk patients via mail-out, which is a novel mechanism. Have adapted to Safer Prescribing and care for the Elderly (SPACE) in NZ – this involves practice audit of NSAID/anticoagulant prescribing, a visit (to GPs) of PHO pharmacist to give education and patient-specific feedback, and a mail out to patients receiving high risk medication combinations prompting discussion with Dr at next visit. Started a pilot in Oct 2016 with 7 GPS from a single PHO and 86 high risk PTs from total of 870. Six month results due in April 2017. Data collection and NHI collection of hospitalisation and prescribing successful in pilot.
Will run 12 month trial targeting high risk NSAID prescribing. Practices will be randomised perpostively to include small and large practices and involvement is approved as continuous quality improvement by college which should increase GP participation. Randomise practices 1:1 intervention to control. The SPACE intervention comprises a practice audit to identify people with high-risk prescribing for NSAIDs and/or antiplatelet medicines amongst 'vulnerable patients' not sure if this mean GPs patients or both); visit by a **community** pharmacist to deliver to doctors patient-specific feedback and educational material; a tick-box for doctors to indicate the action they will take in response to the feedback (presumably they will monitor this but don't say); a mail-out of the educational material to local community pharmacists; and, one month later, a mail-out from the practice to patients identified as having high-risk prescribing containing information about their medicines and a letter encouraging them to
discuss their medicines when they next see their doctor. Main outcome measure rate of high risk prescribing. 2ndry outcomes rates of high risk prescribing in each of the three risk areas (table 1) and event rate and incidence of admissions. This data will enable subsequent cost-effectiveness analysis. Seems to me admission should be the main outcome!
Team Wallis academic GP 24 papers mainly first author. Kerse and Elley great track records. Bryant pharmacist. 14 papers.
Budget $30 for pharmacist to do intervention. $50K Senior RF to do recruitment and help analysis. $26K data analyst and 2K statistician. Costs for SQL???? For identification of vulnerable patients and sending letters etc.

PROJECT GRANTS
Medical Committee Report Form

Auckland Medical Research Foundation
est. 1955

**APPLICANTS**	Wallis
**SHORT TITLE**	SPACE cluster RCT in general practice

Overall Summary / Key Points: Please use the following topics as a guide.

- ☐ Relevance: Is the concept important, and are the aims appropriate and clearly defined?
- ☐ Quality: Is the application well written? Does the experimental approach have scientific merit?
- ☐ Experience (Relative to opportunity): Is the application to a standard you would expect for the PI's career stage? Do the applicants have good track records, and are they capable of achieving the objectives?
- ☐ Other: Are there minor or major issues with the application and/or applicants?

This application for funding for a cluster RCT looking at the effect of an intervention to reduce high risk prescribing in primary care. This project addresses an important clinical issue, and has the potential for a large improvement in health outcomes.

The application is generally clear and well written by a group of investigators with expertise in clinical trials in primary care. They have included data from a pilot study that shows that the core elements of the study are in place.

The budget is for a senior research fellow (maybe the PI??), pharmacist to complete the study intervention, analyst and statistician, data management, consumables.

The timeline includes ethics application over three months (an approval is provided which does not directly relate to the proposed application), then recruitment of 4 practices/month. This means that it will take a total of 10 months to recruit. The budget and timelines do not align with this plan, noting that a pharmacist is only funded for 6 months.

Referees raise concerns that the project is underfunded and the issues with the details of the intervention, sample size and timelines are pertinent.
